# Genetic Variability of Incretin Receptors and Alcohol Dependence: A Pilot Study

**DOI:** 10.3389/fnmol.2022.908948

**Published:** 2022-06-09

**Authors:** Evangelia Eirini Tsermpini, Katja Goričar, Blanka Kores Plesničar, Anja Plemenitaš Ilješ, Vita Dolžan

**Affiliations:** ^1^Pharmacogenetics Laboratory, Institute of Biochemistry and Molecular Genetics, Faculty of Medicine, University of Ljubljana, Ljubljana, Slovenia; ^2^University Psychiatric Clinic, Ljubljana, Slovenia; ^3^Faculty of Medicine, University of Ljubljana, Ljubljana, Slovenia; ^4^Department of Psychiatry, University Clinical Centre Maribor, Maribor, Slovenia

**Keywords:** alcohol dependence, alcohol-related psychosymptomatology, incretin receptors, GIPR, GLP-1R, polymorphism

## Abstract

Alcohol dependence is a chronic mental disorder that leads to decreased quality of life for patients and their relatives and presents a considerable burden to society. Incretin hormones, such as glucose-dependent insulinotropic polypeptide (GIP) and glucagon-like peptide 1 (GLP-1) are endogenous gut-brain peptides, which can travel across the blood-brain barrier and access the nervous system. Their respective receptors, GIPR and GLP-1R, are expressed in the reward-related brain areas and are involved in memory formation and neurogenesis, which results in behavioral changes in rodent models. The current study investigated the potential association of genetic variability of incretin receptors with alcohol dependence and alcohol-related psychosymptomatology. Alcohol dependence and comorbid psychosymptomatology were assessed in a cohort of Slovenian male participants, comprised of 89 hospitalized alcohol-dependent patients, 98 abstinent alcohol-dependent patients, and 93 healthy blood donors. All participants were genotyped for *GIPR* rs1800437 and *GLP1R* rs10305420 and rs6923761 polymorphisms. For the statistical analysis Kruskal–Wall and Mann–Whitney tests were used in additive and dominant genetic models. Our findings indicated that *GIPR* rs1800437 genotypes were associated with an increased risk of alcohol dependence. Statistically significant association between *GIPR* rs1800437 GG genotype and Brief Social Phobia Scale scores were observed in the abstinent alcohol-dependent patients, while *GLP1R* rs6923761 GG genotype was associated with Zung anxiety scores in healthy controls. Our pilot study indicates that *GIPR* rs1800437 may play some role in susceptibility to alcohol dependence, as well as in alcohol-related psychosymptomatology symptoms. To our knowledge, this is the first study that indicates the involvement of *GIPR* in alcohol dependence. However, studies with larger cohorts are needed to confirm these preliminary findings.

## Introduction

Alcohol dependence is a chronic mental disorder characterized by an intense craving for alcohol and the inability to control or stop alcohol consumption, usually accompanied by a history of excessive drinking (Carvalho et al., 2019; Domi et al., 2021). Regarding its epidemiology, alcohol dependence is one of the most prevalent mental disorders worldwide, and it is five times more frequent in men than in women. In addition, alcohol dependence was found to be more frequent in high-income and upper -middle- income countries for both males and females (Carvalho et al., 2019). Alcohol dependence is also associated with high morbidity and mortality rates. Alcohol dependence is also related to other comorbid mental disorders, such as major depressive disorder, anxiety disorders, schizophrenia, bipolar disorder, and attention deficit hyperactivity disorder (Gandal et al., 2018; Kranzler and Soyka, 2018; Walters et al., 2018; Rudenstine et al., 2020; Zhou et al., 2020).

Alcohol dependence leads to decreased quality of life for patients and their relatives and presents a considerable burden to society (Carvalho et al., 2019; Klausen et al., 2022). Alcohol dependence and alcohol abuse used to be separate disorders in the Diagnostic and Statistical Manual of mental disorders (DSM-IV), whereas, in DSM-V, they are integrated into one broader category of alcohol use disorder (AUD) which includes sub-classifications, depending on the severity of the symptoms (Kathryn Mchugh and Weiss, 2019; Nutt et al., 2021). The stages of dependence can be divided into the acute and chronic state, followed by short-term and long-term abstinence. What sets them apart is the duration of each stage and the underlying molecular and cellular mechanisms involved (Nestler and Aghajanian, 1997; Koob and Volkow, 2016).

The emergence and perpetuation of AUD can be due to several factors, including genetic, environmental risk factors, and gene-environment interactions (Nestler and Aghajanian, 1997; Carvalho et al., 2019). Family, twin, and adoption studies (Cloninger et al., 1981; Heath et al., 1997; Verhulst et al., 2015) and a recent meta-analysis (Verhulst et al., 2015) indicated that heritability estimates are pretty high. Preclinical and clinical studies have shown that genetic variability is associated with susceptibility and development of AUD (Hiroi and Agatsuma, 2005; Jones et al., 2015; Bowen et al., 2022). A genome-wide meta-analysis on AUD and problematic alcohol use, which included 435,563 subjects of European ancestry, identified 29 independent risk variants, 19 of which were novel (Zhou et al., 2020).

Glucagon-like peptide 1 (GLP-1) and glucose-dependent insulinotropic polypeptide, also known as gastric inhibitory polypeptide (GIP), are endogenous gut-brain peptides that function both as a hormone and neuropeptide and are released from intestinal L-cells and K-cells, respectively, in response to food intake in humans and mice (Adner and Nygren, 1992; Dalgaard et al., 2004; Alvarez et al., 2005; Pannacciulli et al., 2007; Seino et al., 2010; Seino and Yabe, 2013; Abraham et al., 2015; Jerlhag, 2020; Marty et al., 2020; Eren-Yazicioglu et al., 2021). They stimulate glucose-induced insulin secretion, inhibit glucagon secretion, and decrease appetite in humans and mice (Adner and Nygren, 1992; Dalgaard et al., 2004; Seino et al., 2010; Underwood et al., 2010; Seino and Yabe, 2013; Abraham et al., 2015; Klausen et al., 2022). They have the ability to travel across the blood-brain barrier and access the nervous system in humans and rats (Isbil-Buyukcoskun et al., 2009; Eren-Yazicioglu et al., 2021). GLP-1 and GIP act by binding with their receptors GLP-1R and GIPR, respectively, which are members of the G-protein coupled receptors and are expressed in the peripheral and central nervous system (Seino et al., 2010; Seino and Yabe, 2013). GLP-1 and GIP and their receptors are involved in memory formation and neurogenesis in rats (Seino et al., 2010). GIPR is expressed in neurons, and GLP-1R can be found in several tissues, including human, rat and mice brain regions related to reward and addiction (Merchenthaler et al., 1999; Alvarez et al., 2005; Pannacciulli et al., 2007; Rinaman, 2010; Seino et al., 2010; Suchankova et al., 2015; Jensen et al., 2018; Eser et al., 2020; Eren-Yazicioglu et al., 2021). It can also modulate dopamine levels and glutamatergic neurotransmission, which results in behavioral changes in rats, and mice (Alhadeff et al., 2012; Reddy et al., 2016; Eren-Yazicioglu et al., 2021). Also, it displayed neuroprotective effects in male rats, mice, non-human primates (Klausen et al., 2022) and humans (Erbil et al., 2019). Furthermore, both preclinical and clinical studies indicated the crucial role of GLP-1R in reward function and addictive disorders, including alcohol-seeking and AUD (Egecioglu et al., 2013; Suchankova et al., 2015; Jayaram-Lindström et al., 2016; Marty et al., 2020; Eren-Yazicioglu et al., 2021). Animal model studies have suggested that peptides like GLP-1 regulate behavioral responses to alcohol consumption (Seino et al., 2010; Jerlhag, 2020), however, the role of GIP and its receptor has not been studied so well and the exact mechanism of action is not yet fully known.

GLP-1R also seems to have a neuroprotective role and, thus, has been investigated as a target in the cerebral infarction treatment (Seino and Yabe, 2013). There is also evidence that GLP-1R stimulation regulates alcohol-seeking and wanting behaviors (Suchankova et al., 2015; Eren-Yazicioglu et al., 2021). Nevertheless, based on the mechanism of action of FDA-approved GLP-1 receptor agonists, reduction in alcohol consumption can be due to the discomfort felt during alcohol use and abstinence and reduction in rewarding effects. GLP-1R agonists reduce the rewarding effects of alcohol, which leads to decreased alcohol intake. Exentin-4 is a well know example of GLP-1R agonist that affects the signal transmission, and according to studies, GLP-1R and GIPR have similar molecular mechanisms (Seino et al., 2010).

Genetic variability of GLP-1R and GIPR has been investigated in human pathologies, such as metabolic and cardiovascular diseases, and bone mineral density. According to the literature, genetic variability influences response to incretin peptides and their antagonists (Jensterle et al., 2015; Klen and Dolžan, 2022). More specifically, *GLP1R* rs10305420 has been associated with response to exenatide in overweight patients with type 2 diabetes (Yu et al., 2019) and liraglutide in obese women with polycystic ovary syndrome (Jensterle et al., 2015). Regarding *GLP1R* rs6923761, it has been shown to relate with metabolic and obesity parameters, such as body mass index, weight, fat mass, waist circumference, triglycerides, insulin, HOMA-IR, and HDL cholesterol (de Luis et al., 2013, 2014a,b,c,e, 2015b,d, 2018), weight loss (de Luis et al., 2014a,d), cardiovascular risk in patients with obesity (de Luis et al., 2018), type 2 diabetes (de Luis et al., 2015a). It has also been associated with gliptin therapies, like the DPP-4 inhibitor sitagliptin and vildagliptin (Javorský et al., 2016; Urgeová et al., 2020; Mashayekhi et al., 2021), liraglutide and exenatide (de Luis et al., 2015c; Chedid et al., 2018). *GIPR* rs1800437 has been associated with glucose homeostasis (Sauber et al., 2010), obesity (Vogel et al., 2009), heart failure prognosis in obese patients (Agra et al., 2019), bone mineral density and fracture risk (Torekov et al., 2014).

Glucagon-like peptide 1 variability has been investigated in two preclinical studies with mice AUD models, one of which also included a cohort of AUD patients and controls (Koole et al., 2011; Suchankova et al., 2015). However, to our knowledge, there are no studies that focus on *GIPR* polymorphisms and alcohol.

The current study aimed to investigate the potential association of *GLP1R* rs10305420 and rs6923761 and *GIPR* rs1800437 with alcohol dependence, as well as alcohol-related comorbid psychosymptomatology.

## Materials and Methods

### Study Population

The study cohort included three groups of participants: hospitalized alcohol-dependent patients, abstinent alcohol-dependent patients, and healthy controls with no alcohol dependence history. All participants were male of Slovenian origin, aged 18 to 66. Experienced psychiatrists recruited patients hospitalized for treatment of alcohol dependence at the University Clinical Center Maribor and the University Psychiatric Clinic Ljubljana. The inclusion criteria for the hospitalized alcohol-dependent patients were a diagnosis of alcohol-dependence, according to the DSM-IV (American Psychiatric Association, 2000), with no significant symptoms of abstinence, after hospitalization for at least 2 weeks. The abstinent alcohol-dependent patients were recruited from support group meetings, and the inclusion criterion was abstinence for at least 2 years. Exclusion criteria for these two groups of patients were: a medical history of mental or neurological disorders or significant medical conditions and a previous diagnosis of dependence (nicotine not included) according to DSM-IV. The healthy controls were blood donors with no DSM-IV axis I mental disorders or alcohol consumption problems. The study was approved by the Slovenian National Medical Ethics committee (approval No. 117/06/10 and 148/02/1011).

Written informed consent was obtained from all the participants after they were informed about the scope of the study. At the baseline, all the demographic and clinical data of each patient were also recorded. Demographic variables included age, residence, marital status, academic years, and smoking status. In addition, questionnaires that evaluate comorbid psychosymptomatology were employed in all groups of participants. More specifically, depression and anxiety symptoms were accessed using the Zung Depression (Zung, 1965) and Anxiety (Zung, 1971) scale, social anxiety symptoms, using the Brief Social Phobia Scale (BSPS) (Davidson et al., 1997), drinking habits, and severity of alcohol use and dependence using the Alcohol Use Disorders Identification Test (AUDIT) (Rush et al., 2008), obsessive-compulsive traits, using the Yale-Brown Obsessive-Compulsive Scale (YBOCS) (Goodman and Price, 1992) and Obsessive-Compulsive Drinking Scale (OCDS) (Anton, 2000), and symptoms of aggression and hostility, were evaluated using the and the Buss-Durkee Hostility Inventory (BDHI) (Buss and Durkee, 1957). More information about the cohorts can be found in our previous articles (Plemenitas et al., 2015; Ilješ et al., 2021).

### Molecular Genetic Analysis

DNA was extracted from whole blood for hospitalized alcohol-dependent patients and healthy controls, whereas DNA was extracted from buccal swabs for the abstinent alcohol-dependent group of patients. QIAamp Blood Mini kit was used for the DNA extraction from whole blood, collected using ethylenediaminetetraacetic acid (EDTA) and QIAamp Mini kit for the DNA extraction from buccal swabs, according to the manufacturer’s protocols (Qiagen GmbH, Hilden, Germany).

Genotyping was performed using fluorescence-based competitive allele-specific PCR (KASP) amplification combining KASP Master mix and custom validated KASP Genotyping Assays with a KASP reporting system, according to the manufacturer’s instructions (LGC Genomics, United Kingdom). Thermal cycling conditions are presented in Supplementary Table 1.

### Statistical Analysis

The statistical analyses were performed with IBM SPSS Statistics, version 27.0 (IBM Corporation, Armonk, NY, United States). The cut-off for the statistical significance was set at 0.05. Pearson’s chi-square test was used to assess deviation from Hardy–Weinberg equilibrium (HWE) in healthy individuals for all studied polymorphisms. Additive and dominant genetic models were used in the analysis. To compare clinical characteristics between patient groups, we used Fisher’s exact test for categorical variables and the Kruskal–Wallis test with 2 degrees of freedom for continuous variables. Fisher’s exact test was also used to compare the frequencies of the rs10305420, rs6923761, and rs1800437 between the three studied groups. In logistic regression, odds ratios (ORs) and 95% confidence intervals (CIs) were determined. Age, residence place, marital status, academic years, and smoking status were considered as covariates and significant variables were used for adjustment in regression analysis. The association of genotypes with psychosymptomatology scores was evaluated using the Kruskal–Wallis and Mann–Whitney non-parametric tests for additive and dominant genetic models, respectively.

## Results

Our cohort comprised of 89 hospitalized alcohol-dependent patients, 98 abstinent alcohol-dependent patients, and 93 healthy controls with no alcohol dependence history (Table 1). Regarding the demographic characteristics, the median age of the hospitalized alcohol-dependent and abstinent alcohol-dependent patients was significantly higher compared to healthy controls (*p* < 0.001). The distribution of the years of education also differed among groups (*p* < 0.001), but there were no differences in residence place (*p* = 0.265). However, the majority of healthy controls and abstinent alcohol-dependent patients were smokers (*p* < 0.001) and had a partner (*p* = 0.005) in comparison with hospitalized alcohol-dependent patients (Table 1).

**TABLE 1:** Cohort’s characteristics.

Characteristic		Healthy controls (*N* = 93)	Abstinent alcohol-dependent (*N* = 98)	Hospitalized alcohol-dependent (*N* = 89)	*P* *
Age	Years, median (25–75%)	36 (26–44.5)	49 (44–54.3)	47 (39–54)	<0.001
Education	Years, median (25–75%)	12 (12–12)	12 (11–12)	12 (11–12)	<0.001
Partnership	Single, *N* (%)	25 (26.9)	21 (21.4)	38 (42.7)	0.005
	Partnership, *N* (%)	68 (73.1)	77 (78.6)	51 (57.3)	
Environment	Rural, *N* (%)	37 (39.8)	46 (46.9)	46 (51.7)	0.265
	Urban, *N* (%)	56 (60.2)	52 (53.1)	43 (48.3)	
Smoking	No, *N* (%)	24 (25.8)	48 (49.0)	58 (65.2)	<0.001
	Yes, *N* (%)	69 (74.2)	50 (51.0)	31 (34.8)	

*Calculated using Fisher’s exact test for categorical variables and Kruskal–Wallis test for continuous variables.

Regarding the questionnaires, differences were observed between the three groups in the scores of Zung Depression and Anxiety scale, YBOCS obsession and compulsion scale, AUDIT, OCDS, and BDHI questionnaires (all *p* < 0.05), but not for BSPS (*p* = 0.623) (Table 2 and Supplementary Table 2).

**TABLE 2:** Questionnaire scores.

Questionnaire		Healthy controls (*N* = 93)	Abstinent alcohol-dependent (*N* = 98)	Hospitalized alcohol-dependent (*N* = 89)	*P* *
YBOCS obsession	Points, median (25–75%)	1 (1–1)	1 (1–1.3)	2 (1–7)	<0.001
YBOCS compulsion	Points, median (25–75%)	1 (1–1)	1 (1–1)	1 (1–3)	<0.001
BSPS	Points, median (25–75%)	9 (5.5–14)	10 (5–18.3)	10 (4–18.5)	0.623
AUDIT	Points, median (25–75%)	5 (4–7)	3 (3–5)	23 (19–28.5)	<0.001
OCDS	Points, median (25–75%)	3 (2–4)	2 (2–3)	18 (9–26.5)	<0.001
Zung depression	Points, median (25–75%)	22 (20–24)	29 (25–35)	34 (27–45)	<0.001
Zung anxiety	Points, median (25–75%)	22 (20–24)	28 (25–35)	34 (29–39)	<0.001
BDHI	Points, median (25–75%)	17 (10.5–23)	24 (15.8–31)	30 (22–40)	<0.001

*Calculated using Kruskal–Wallis test.

The genotype distributions for all the studied polymorphisms were in HWE for the healthy controls (GG: 65.6%, GC: 26.9%, CC: 7.5%; *p* = 0.068 for rs1800437, CC: 51.1%, CT: 37.8%, TT: 11.1%; *p* = 0.340 for rs10305420 and GG: 46.2%, GA: 45.2%, AA: 8.6%; *p* = 0.614 for rs6923761).

When comparing all three groups, no differences in the distribution of genotype frequencies were observed for any of the studied polymorphisms (*p* = 0.155 for rs1800437; *p* = 0.645 for rs10305420 and *p* = 0.632 for rs6923761) (Supplementary Table 3).

Given that both the groups of hospitalized and abstinent patients had the diagnosis of alcohol dependence, we merged these two groups into one and we compared the genotype frequencies with those of the healthy controls, an association was observed for *GIPR* rs1800437 GC (OR = 1.77, 95% CI = 1.02–3.09, *p* = 0.043) and GC + CC genotypes (OR = 1.69, 95% CI = 1.01–2.84, *p* = 0.045), but it did not remain statistically significant after adjustment for age, education, smoking, and partnership. No associations were observed for *GLP1R* polymorphisms (Table 3).

**TABLE 3:** Comparison of genotype frequencies between all alcohol-dependent patients and healthy controls.

Gene	SNP	Genotype	OR (95% CI)	*P*	OR (95% CI) adj	Padj
*GIPR*	rs1800437	GG	Reference		Reference	
		GC	1.77 (1.02–3.09)	**0.043**	1.71 (0.85–3.44)	0.135
		CC	1.41 (0.55–3.62)	0.477	1.79 (0.59–5.46)	0.303
		GC + CC	1.69 (1.01–2.84)	**0.045**	1.73 (0.90–3.31)	0.100
*GLP1R*	rs10305420	CC	Reference		Reference	
		CT	1.07 (0.63–1.82)	0.809	1.24 (0.62–2.47)	0.548
		TT	1.44 (0.64–3.23)	0.374	1.26 (0.46–3.47)	0.653
		CT + TT	1.15 (0.70–1.89)	0.583	1.24 (0.66–2.35)	0.505
*GLP1R*	rs6923761	GG	Reference		Reference	
		GA	0.96 (0.57–1.61)	0.866	1.13 (0.58–2.21)	0.721
		AA	0.65 (0.24–1.73)	0.389	0.38 (0.11–1.31)	0.124
		GA + AA	0.91 (0.55–1.49)	0.702	0.96 (0.51–1.82)	0.910

*Adj: adjusted for age, education, smoking, and partnership. Statistically significant p values are printed in bold.*

We also compared each group of alcohol-dependent patients with the controls separately. When comparing genotype frequencies between abstinent alcohol-dependent patients and healthy controls, no statistically significant difference was observed for any of the three studied polymorphisms, neither before nor after adjustments for age, education and smoking (Supplementary Table 4).

However, *GIPR* rs1800437 GC + CC and GC genotypes were significantly more frequent in hospitalized alcohol-dependent patients than in healthy controls (OR = 2.13, 95% CI = 1.17–3.87, *p* = 0.013 and OR = 2.21, 95% CI = 1.16–4.19, *p* = 0.015, respectively). The association remained statistically significant for GC + CC genotypes in the dominant model after adjustment for age, education, smoking, residence, and partnership (OR = 2.42, 95% CI = 1.07–5.48, *p* = 0.035). No significant differences in *GLP1R* genotype frequencies’ distribution were observed between these two groups (Table 4).

**TABLE 4:** Comparison of genotype frequencies between hospitalized alcohol-dependent patients and healthy controls.

Gene	SNP	Genotype	OR (95% CI)	*P*	OR (95% CI) adj	Padj
*GIPR*	rs1800437	GG	Reference		Reference	
		GC	2.21 (1.16–4.19)	**0.015**	2.15 (0.90–5.15)	0.087
		CC	1.87 (0.65–5.41)	0.250	3.69 (0.93–14.70)	0.064
		GC + CC	2.13 (1.17–3.87)	**0.013**	2.42 (1.07–5.48)	**0.035**
*GLP1R*	rs10305420	CC	Reference		Reference	
		CT	1.08 (0.57–2.03)	0.818	1.20 (0.50–2.88)	0.680
		TT	1.88 (0.77–4.60)	0.167	1.50 (0.43–5.20)	0.524
		CT + TT	1.25 (0.70–2.24)	0.450	1.27 (0.57–2.85)	0.556
*GLP1R*	rs6923761	GG	Reference		Reference	
		GA	0.93 (0.51–1.70)	0.820	0.66 (0.29–1.55)	0.343
		AA	0.36 (0.09–1.44)	0.148	0.23 (0.04–1.41)	0.112
		GA + AA	0.84 (0.47–1.51)	0.560	0.58 (0.26–1.30)	0.186

*Adj: adjusted for age, education, smoking, residence, and partnership. Statistically significant p values are printed in bold.*

**FIGURE 1:**
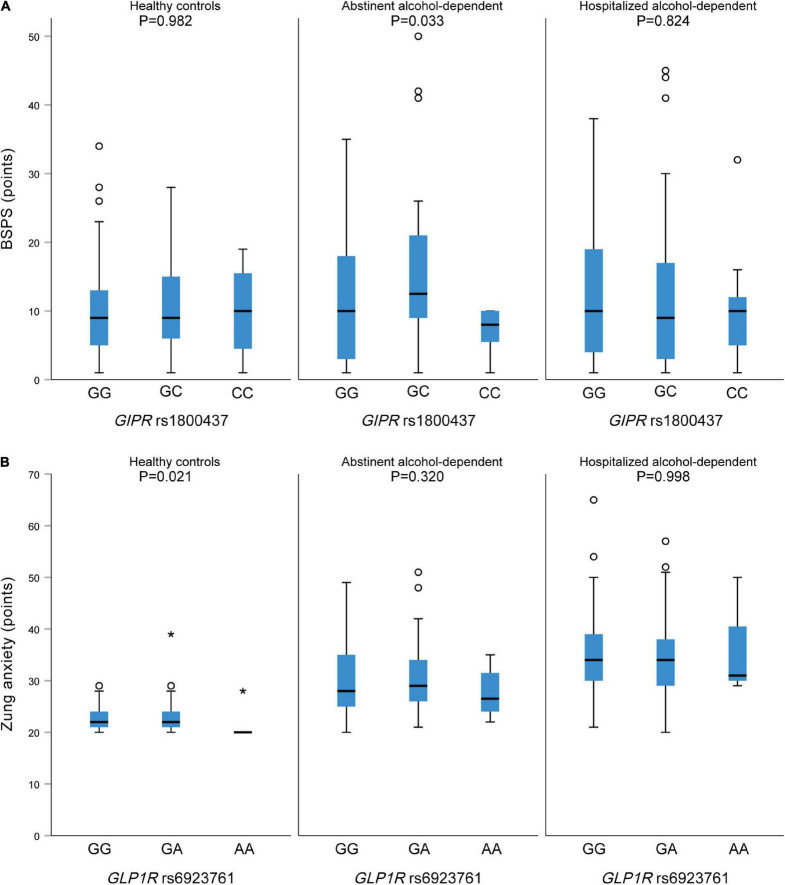
**(A)** Association between GIPR rs1800437 and BSPS score in healthy controls (left), abstinent (middle) and hospitalized alcohol-dependent (right) patients. **(B)** Association between GLP1R rs6923761 and Zung anxiety score in healthy controls (left), abstinent (middle) and hospitalized alcohol-dependent (right) patients.

Regarding the potential relation between the studied polymorphisms and psychosymptomatology scores, we observed a statistically significant association between *GIPR* rs1800437 CC genotype and lower BSPS scores in the abstinent alcohol-dependent patients (*p* = 0.033) (Figure 1A). *GIPR* genotypes were not associated with any of the other psychosymptomatology scores (Table 5).

**TABLE 5:** Associations between *GIPR* rs1800437 and the assessed psychosymptomatology scores.

Scale	*GIPR* rs1800437 genotype	Healthy controls (*N* = 93)		Abstinent alcohol-dependent (*N* = 98)		Hospitalized alcohol-dependent (*N* = 89)	
				
		Median (25–75%)	*P* *	Median (25–75%)	*P* *	Median (25–75%)	*P* *
YBOCS obsession	GG	1 (1–1)	0.880	1 (1–2)	0.227	2 (1–7)	0.129
	GC	1 (1–1.5)		1 (1–1)		1 (1–8.3)	
	CC	1 (1–4)		1 (1–2)		1 (1–1.5)	
	GC + CC	1 (1–1.8)	0.991	1 (1–1)	0.118	1 (1–7)	0.228
YBOCS compulsion	GG	1 (1–1)	0.653	1 (1–1)	0.098	1 (1–6)	0.213
	GC	1 (1–1)		1 (1–1)		1 (1–2.3)	
	CC	1 (1–2)		1 (1–2)		1 (1–1)	
	GC + CC	1 (1–1)	0.677	1 (1–1)	0.315	1 (1–2)	0.356
BSPS	GG	9 (5–13.5)	0.982	10 (3–18)	**0.033**	10 (4–19)	0.824
	GC	9 (6–16)		12.5 (8.8–21.5)		9 (3–17.5)	
	CC	10 (3–18)		8 (3–10)		10 (3–14)	
	GC + CC	9 (6–16.5)	0.894	11 (8–20)	0.118	9 (3–17)	0.714
AUDIT	GG	5 (4–7)	0.194	3 (3–4)	0.999	23 (18.8–29.3)	0.208
	GC	4 (3–6)		3 (3–5)		23 (19.8–28)	
	CC	6 (5–8)		3 (3–5)		27 (22.5–32.5)	
	GC + CC	5 (3–6)	0.297	3 (3–5)	0.959	23 (20–28)	0.792
OCDS	GG	3 (2–4)	0.444	2 (2–3)	0.885	16 (9.8–26)	0.889
	GC	3 (2–4)		2 (2–3.3)		18 (8.8–29)	
	CC	4 (2–5)		2 (2–4)		19 (8–24.5)	
	GC + CC	3 (2–4)	0.535	2 (2–4)	0.792	18 (8–28)	0.736
Zung depression	GG	22 (20.5–24.5)	0.493	28 (24–34)	0.252	36 (25–49)	0.550
	GC	22 (20–22.5)		29.5 (27–35.3)		31.5 (27–40.5)	
	CC	21 (20–26)		32 (29–35)		35 (29–42)	
	GC + CC	22 (20–22.8)	0.251	31 (27–35)	0.106	32 (28–40)	0.300
Zung anxiety	GG	22 (20.5–25)	0.484	28 (25–32.5)	0.202	34.5 (29–42.5)	0.491
	GC	21 (20–23)		30 (26–37.3)		33.5 (29.8–37.3)	
	CC	21 (20–26)		30 (23–35)		32 (27–39.5)	
	GC + CC	21 (20–23)	0.238	30 (26–36.5)	0.078	33 (29–38)	0.319
BDHI	GG	17 (12–24.5)	0.483	24 (17–29)	0.420	33.5 (19.8–40)	0.080
	GC	13 (8–22.5)		25.5 (14.8–33)		33 (25.8–42.3)	
	CC	20 (11–22)		21 (14–25)		22 (15.5–29.5)	
	GC + CC	14.5 (8–22)	0.369	24 (14–31)	0.759	29 (24–42)	0.793

*Kruskal–Wall test for additive and Mann–Whitney test for the dominant model. Statistically significant p values are printed in bold.

No statistically significant associations were observed between *GLP1R* rs10305420 and the assessed psychosymptomatology scores in any of the study groups (data not shown). However, *GLP1R* rs6923761 AA genotype was associated with lower Zung anxiety scores among healthy controls (*p* = 0.021) (Figure 1B and Supplementary Table 5).

## Discussion

We conducted a pilot study to investigate the role of *GLP1R* rs10305420 and rs6923761 and *GIPR* rs1800437 in alcohol dependence and related psychosymptomatology in a cohort of hospitalized alcohol-dependent patients, abstinent alcohol-dependent patients, and healthy individuals. To our knowledge, this is the first study that focuses on the role of *GIPR* on alcohol dependence and one of a few that investigated the relation of *GLP1R* with alcohol dependence in humans (Koole et al., 2011; Suchankova et al., 2015).

According to our results, *GIPR* rs1800437 genotypes were associated with an increased risk of alcohol dependence. No statistically significant associations were found for *GLP1R* rs10305420 and rs6923761 with alcohol dependence. We also observed statistically significant association between *GIPR* rs1800437 GG genotype and BSPS scores in the abstinent alcohol-dependent patients as well as the association between *GLP1R* rs6923761 GG genotype and Zung anxiety scores in healthy controls.

It is crucial to mention that this is the first study that indicates the involvement of GIPR in alcohol dependence and alcohol-related comorbid psychosymptomatology. The potential participation of GIP and its receptor in the etiology and pathophysiology of alcohol is limited. We know that GIPR is expressed in the adult rat hippocampus, a brain region related to memory (Nyberg et al., 2005). An animal model study has shown that mice with GIPR deficiency have synaptic plasticity deterioration, impaired neurogenesis, and learning disabilities (Faivre et al., 2011). Interestingly, GIP regulates progenitor cell proliferation (Nyberg et al., 2005) and neurotransmitter release and has a protective role on the synapses during synaptic plasticity (Gault and Hölscher, 2008). Alcohol use impacts the activity of the synapses, i.e., the points of contact between neurons, which affects the transmission of the information from one neuron to the next (Nestler and Aghajanian, 1997). Further studies are therefore needed to elucidate the role of GIPR genetic variability in AUD.

Regarding GLP-1, we observed an association between *GLP1R* rs6923761 GG genotype and Zung anxiety scores in healthy controls, but not between GLP1R rs10305420 and rs6923761 and alcohol dependence. Our results are in contrast with the findings of preclinical and clinical studies. An animal study indicated an interaction between alcohol use and the GLP-1 system, which might further elucidate the role of GLP-1R containing brain areas in reducing alcohol reinforcement through GLP-1R agonists and support the usage of GLP-1R as potential treatment targets for AUD. More specifically, the expression of the GLP-1 receptor in nucleus accumbens, which is the neural interface between motivation and action, was increased in high alcohol-consuming rodents compared to those under low alcohol consumption (Vallöf et al., 2019). In addition, Suchankova et al. (2015) investigated the impact of GLP-1R genetic variability on AUD in humans and a mouse model. Initially, they performed a case-control study that included 670 AUD patients and 238 controls with no current or past alcohol abuse. Then, the emerged significant associations were examined on a genome-wide association cohort of 1,917 patients with alcohol dependence and 1,886 healthy individuals. For functional validation of the findings, they included 84 participants who underwent intravenous self-administration of alcohol. To evaluate brain activity changes, they performed functional magnetic resonance imaging (fMRI) in 22 patients with alcohol dependence. Finally, they investigated the impact of GLP-1R agonism on alcohol dependence in a mouse model. Overall, their results indicated that the rs6923761 A (Ser) allele was nominally associated with increased AUD. Also, rs6923761 heterozygotes had higher alcohol self-administration and higher Blood-oxygen-level-dependent imaging (BOLD) signal in the globus pallidus when participants received rewarding outcomes during the Monetary Incentive Delay task. Lastly, from the preclinical model emerged a significant reduction of alcohol use after pharmacological GLP-1R agonist (Suchankova et al., 2015). An earlier *in vitro* study has also shown that rs6923761 has a functional role, given that the A (Ser) allele is associated with reduced GLP-1R expression levels in the cell’s surface (Koole et al., 2011). In our study, only potential association with psychosymptomatology was observed for rs6923761, while no differences in genotype frequencies were observed among different groups.

Regarding GLP-1 and its influences on reward processing through globus pallidus, ventral tegmental area and nucleus accumbens could explain detected association of *GIPR* rs1800437 and social anxiety scores, since neural activation in globus pallidus, among others, is associated with social phobia and anxiety disorders (Hattingh et al., 2013; Suchankova et al., 2015; Ashworth et al., 2021). Decreased connectivity between the nucleus accumbens and putamen was also reported in connection with social anxiety disorder. To the best of our knowledge, no human study explored the association of GLP-1 and GIP on the expression of anxiety symptoms. GLP-1 receptor gene polymorphism rs1042044 was associated with anhedonia, a symptom of major depressive disorder (Eser et al., 2020). Another human study reported about abnormal gene expression of GLP-1R in post-mortem brain of individuals with mood disorder (Mansur et al., 2019). The gut-brain axis with gastrointestinally derived neuropeptides like GLP-1, are emerging as potential key regulators of anxiety behavior. A study performed on rats reported chemogenetic activation of neurons and anxiolytic response. Another animal studies on rats reported anxiogenic and antidepressant effects of GLP-1 receptor stimulation and anti-anxiety effect of liraglutide which is GLP-1 agonist (Sharma et al., 2015; Anderberg et al., 2016). So further studies on this are warranted to elucidate this issue.

Nevertheless, our study has some limitations, such as the small sample size, and the lack of data on metabolic parameters of the participants. Given that age and the proportion of smokers differed between the three studied groups, differences in subjects’ characteristics were considered as an adjustment in logistic regression analysis. Another limitation is the inclusion of a cohort comprised only of male participants. However, it should be noted that all animal model studies focus on male animals, and the innovative research of Suchankova mentioned above also indicated that the association between AUD and GLP-1R was more significant in men (Suchankova et al., 2015). In a mice AUD model, it has been shown that males and females are different in terms of alcohol consumption and response during the potential forced abstinence, which is known to affect interconnected networks of neural circuits that are associated with depression and anxiety symptoms. Female mice consumed more alcohol, but they could transit to an abstinence-induced depressive state more quickly than male mice (Dao et al., 2020). Murano et al. (2017) found a similar pattern of gene activity in the hippocampus and the prefrontal cortex of men with alcoholism and infants’ developing brains, but not with women. These brain regions are associated with memory deficiency and cognitive problems, which are also symptoms of patients with alcoholism. They concluded that it is possible that these two brain region alterations are associated with the predisposition of patients to the alcohol abuse (Murano et al., 2017).

Concluding, our pilot study revealed a potential association between *GIPR* and alcohol dependence. Confirming this association in studies with bigger sample sizes and deciphering the role of genetic susceptibility may help with the identification of high-risk individuals and may also open the way to conceive novel treatment strategies.

## Data Availability Statement

The original contributions presented in the study are included in the article/Supplementary Material, further inquiries can be directed to the corresponding authors.

## Ethics Statement

This study involving human participants was reviewed and approved by the Slovenian National Medical Ethics committee. The patients/participants provided their written informed consent to participate in this study.

## Author Contributions

EET, API, and VD: conceptualization. EET, KG, BKP, API, and VD: methodology and writing – review and editing. EET, KG, and API: formal analysis and visualization. KG: statistical analysis. EET and API: writing – original draft preparation. API and VD: supervision. VD: funding acquisition. All authors have read and agreed to the published version of the manuscript.

## Conflict of Interest

The authors declare that the research was conducted in the absence of any commercial or financial relationships that could be construed as a potential conflict of interest.

## Publisher’s Note

All claims expressed in this article are solely those of the authors and do not necessarily represent those of their affiliated organizations, or those of the publisher, the editors and the reviewers. Any product that may be evaluated in this article, or claim that may be made by its manufacturer, is not guaranteed or endorsed by the publisher.
