# Supplementary material for: Genetic Variability of Incretin Receptors and Alcohol Dependence: A Pilot Study
Source: Front Mol Neurosci. 2022 Jun 9;15:908948. doi: 10.3389/fnmol.2022.908948 (PMC9218814; doi:10.3389/fnmol.2022.908948)
Supplement: Supplementary file 1 [file Data_Sheet_1.docx]

Supplementary Material

Supplementary Table 1: Thermal cycling conditions used for *GIPR* rs1800437, *GLP1R* rs6923761, and *GLP1R* rs10305420 genotyping.

| ***GIPR* rs1800437, *GLP1R* rs6923761** | **61-55°C Touchdown protocol** | | |
| --- | --- | --- | --- |
| **Stage** | **Temperature** | **Duration** | **Number of cycles** |
| Hot-start Taq activation | 94°C | 15 min | 1 |
| Touchdown | 94°C | 20 s | 10 |
|  | 61°C (61°C decreasing 0.6°C per cycle to achieve a final annealing / extension temperature of 55°C) | 60 s |  |
| Amplification | 94°C | 20 s | 30 |
|  | 55°C | 60 s |  |
| Read stage | 30°C | 60 s | 1 |
| ***GLP1R* rs10305420** | **68-62°C Touchdown protocol** | | |
| **Stage** | **Temperature** | **Duration** | **Number of cycles** |
| Hot-start Taq activation | 94°C | 15 min | 1 |
| Touchdown | 94°C | 20 s | 10 |
|  | 68°C (68°C decreasing 0.6°C per cycle to achieve a final annealing / extension temperature of 62°C) | 60 s |  |
| Amplification | 94°C | 20 s | 30 |
|  | 62°C | 60 s |  |
| Read stage | 30°C | 60 s | 1 |

Supplementary Table2. Questionnaire categories.

| **Questionnaire** | **Category** | **Healthy controls**  **(N=93)** | **Abstinent alcohol-dependent (N=98)** | **Hospitalized alcohol-dependent (N=89)** | **P*** |
| --- | --- | --- | --- | --- | --- |
| YBOCS obsession | <8 | 92 (98.9) | 94 (95.9) | 71 (79.8) | <0.001 |
|  | ≥8 | 1 (1.1) | 4 (4.1) | 18 (20.2) |  |
| YBOCS compulsion | <8 | 93 (100.0) | 96 (98.0) | 79 (88.8) | <0.001 |
|  | ≥8 | 0 (0.0) | 2 (2.0) | 10 (11.2) |  |
| BSPS | <20 | 84 (90.3) | 75 (76.5) | 72 (80.9) | 0.033 |
|  | ≥20 | 9 (9.7) | 23 (23.5) | 17 (19.1) |  |
| Zung depression | <50 | 93 (100.0) | 96 (98.0) | 77 (86.5) | <0.001 |
|  | ≥50 | 0 (0.0) | 2 (2.0) | 12 (13.5) |  |
| Zung anxiety | <45 | 93 (100.0) | 94 (95.9) | 76 (85.4) | <0.001 |
|  | ≥45 | 0 (0.0) | 4 (4.1) | 13 (14.6) |  |

* calculated using Fisher’s exact test

Supplementary Table 3. Comparison of genotype frequencies among groups.

| **Gene** | **SNP** | **Genotype** | **Healthy controls**  **(N=93)** | **Abstinent alcohol-dependent (N=98)** | **Hospitalized alcohol-dependent (N=89)** | **P*** |
| --- | --- | --- | --- | --- | --- | --- |
| *GIPR* | rs1800437 | GG | 61 (65.6) | 57 (58.2) | 42 (47.2) | 0.155 |
|  |  | GC | 25 (26.9) | 34 (34.7) | 38 (42.7) |  |
|  |  | CC | 7 (7.5) | 7 (7.1) | 9 (10.1) |  |
| *GLP1R* | rs10305420 | CC | 47 (50.5) | 48 (49.0) | 40 (44.9) | 0.645 |
|  |  | CT | 36 (38.7) | 39 (39.8) | 33 (37.1) |  |
|  |  | TT | 10 (10.8) | 11 (11.2) | 16 (18.0) |  |
| *GLP1R* | rs6923761 | GG | 43 (46.2) | 46 (46.9) | 45 (50.6) | 0.632 |
|  |  | GA | 42 (45.2) | 44 (44.9) | 41 (46.1) |  |
|  |  | AA | 8 (8.6) | 8 (8.2) | 3 (3.4) |  |

*Calculated using Fisher’s exact test

Supplementary Table 4. Comparison of genotype frequencies between abstinent alcohol-dependent patients and healthy controls.

| **Gene** | **SNP** | **Genotype** | **OR (95% CI)** | **P** | **OR (95% CI)adj** | **Padj** |
| --- | --- | --- | --- | --- | --- | --- |
| *GIPR* | rs1800437 | GG | Reference |  | Reference |  |
|  |  | GC | 1.46 (0.78-2.73) | 0.243 | 1.45 (0.65-3.25) | 0.360 |
|  |  | CC | 1.07 (0.35-3.24) | 0.905 | 1.37 (0.37-5.10) | 0.638 |
|  |  | GC+CC | 1.37 (0.76-2.47) | 0.292 | 1.44 (0.68-3.03) | 0.342 |
| *GLP1R* | rs10305420 | CC | Reference |  | Reference |  |
|  |  | CT | 1.06 (0.58-1.94) | 0.849 | 1.27 (0.58-2.80) | 0.553 |
|  |  | TT | 1.08 (0.42-2.77) | 0.878 | 1.07 (0.34-3.38) | 0.911 |
|  |  | CT+TT | 1.06 (0.6-1.88) | 0.830 | 1.22 (0.59-2.51) | 0.599 |
| *GLP1R* | rs6923761 | GG | Reference |  | Reference |  |
|  |  | GA | 0.98 (0.54-1.77) | 0.945 | 1.46 (0.67-3.21) | 0.345 |
|  |  | AA | 0.93 (0.32-2.71) | 0.901 | 0.48 (0.13-1.76) | 0.268 |
|  |  | GA+AA | 0.97 (0.55-1.72) | 0.923 | 1.19 (0.57-2.47) | 0.649 |

Adj: adjusted for age, education, and smoking

Supplementary Table 5. Associations between *GLP1R* rs6923761 and the assessed psychosymptomatology scores.

| **Scale** | ***GLP1R r*s6923761** **genotype** | **Healthy controls**  **(N=93)** | | **Abstinent alcohol-dependent (N=98)** | | **Hospitalized alcohol-dependent (N=89)** | |
| --- | --- | --- | --- | --- | --- | --- | --- |
|  |  | Median (25-75%) | P* | Median (25-75%) | P* | Median (25-75%) | P* |
| YBOCS obsession | GG | 1 (1-2) | 0.582 | 1 (1-2) | 0.710 | 2 (1-7.5) | 0.146 |
|  | GA | 1 (1-1) |  | 1 (1-1.8) |  | 1 (1-3.5) |  |
|  | AA | 1 (1-1) |  | 1 (1-1) |  | 7 (1-) |  |
|  | GA+AA | 1 (1-1) | 0.450 | 1 (1-1) | 0.548 | 1 (1-4.8) | 0.114 |
| YBOCS compulsion | GG | 1 (1-1) | 0.350 | 1 (1-1) | 0.438 | 1 (1-4) | 0.563 |
|  | GA | 1 (1-1) |  | 1 (1-1) |  | 1 (1-2.5) |  |
|  | AA | 1 (1-1) |  | 1 (1-1) |  | 3 (1-) |  |
|  | GA+AA | 1 (1-1) | 0.314 | 1 (1-1) | 0.248 | 1 (1-3) | 0.559 |
| BSPS | GG | 10 (6-15) | 0.342 | 11 (5.8-20.3) | 0.773 | 12 (3.5-19) | 0.296 |
|  | GA | 8 (4.8-13) |  | 10 (3.3-17.8) |  | 8 (4-17) |  |
|  | AA | 5.5 (1.5-16.3) |  | 8 (4.3-21.3) |  | 17 (10-) |  |
|  | GA+AA | 8 (4-13) | 0.237 | 10 (4-17.8) | 0.476 | 9 (4-17) | 0.382 |
| AUDIT | GG | 5 (4-6) | 0.933 | 3 (3-5) | 0.529 | 26 (20-29.5) | 0.335 |
|  | GA | 5 (3-7) |  | 3 (3-3) |  | 23 (17.5-28) |  |
|  | AA | 5 (2.3-7) |  | 3 (1.5-4.5) |  | 20 (19-) |  |
|  | GA+AA | 5 (3-7) | 0.717 | 3 (3-3) | 0.293 | 23 (18.3-27.8) | 0.229 |
| OCDS | GG | 3 (2-4) | 0.936 | 2 (2-4) | 0.293 | 18 (9.5-27.5) | 0.836 |
|  | GA | 3 (2-4) |  | 2 (2-3) |  | 16 (8-25) |  |
|  | AA | 3 (2-4) |  | 2 (2-2) |  | 18 (14-) |  |
|  | GA+AA | 3 (2-4) | 0.722 | 2 (2-3) | 0.197 | 17 (8.3-25.5) | 0.608 |
| Zung depression | GG | 22 (21-25) | 0.088 | 30 (24.8-35) | 0.711 | 35 (28.5-43) | 0.732 |
|  | GA | 22 (20-24) |  | 28.5 (26-34.8) |  | 32 (25-47) |  |
|  | AA | 20.5 (20-21) |  | 28 (24.5-31.8) |  | 31 (30-) |  |
|  | GA+AA | 22 (20-24) | 0.402 | 28.5 (26-34) | 0.671 | 31.5 (25-47.5) | 0.516 |
| Zung anxiety | GG | 22 (21-24) | **0.021** | 28 (25-35) | 0.320 | 34 (29.5-39) | 0.998 |
|  | GA | 22 (21-24.3) |  | 29 (26-34.5) |  | 34 (29-38) |  |
|  | AA | 20 (20-20) |  | 26.5 (24-32.3) |  | 31 (29-) |  |
|  | GA+AA | 21 (20-23.3) | 0.249 | 29 (26-33) | 0.526 | 33.5 (29-38) | 0.971 |
| BDHI | GG | 18 (10-23) | 0.458 | 23 (15-31.3) | 0.320 | 34 (23-40) | 0.798 |
|  | GA | 16 (10.8-24.5) |  | 25 (16.5-31) |  | 30 (21.5-41.5) |  |
|  | AA | 13.5 (5-20.8) |  | 18.5 (14.3-25.3) |  | 25 (23-) |  |
|  | GA+AA | 15 (10.8-23.3) | 0.436 | 24.5 (16-30.8) | 0.800 | 30 (22-41) | 0.882 |

*Kruskal-Wall test for additive and Mann-Whitney test for dominant model
